# Multi-locus genome-wide association studies reveal genomic regions and putative candidate genes associated with leaf spot diseases in African groundnut (*Arachis hypogaea* L.) germplasm

**DOI:** 10.3389/fpls.2022.1076744

**Published:** 2023-01-05

**Authors:** Richard Oteng-Frimpong, Benjamin Karikari, Emmanuel Kofi Sie, Yussif Baba Kassim, Doris Kanvenaa Puozaa, Masawudu Abdul Rasheed, Daniel Fonceka, David Kallule Okello, Maria Balota, Mark Burow, Peggy Ozias-Akins

**Affiliations:** ^1^ Groundnut Improvement Program, Council for Scientific and Industrial Research (CSIR)-Savanna Agricultural Research Institute, Tamale, Ghana; ^2^ Department of Agricultural Biotechnology, Faculty of Agriculture, Food and Consumer Sciences, University for Development Studies, Tamale, Ghana; ^3^ Centre d’Etude Régional pour l’Amélioration de l’Adaptation àla Sécheresse (CERAAS), Institut Sénégalais de Recherches Agricoles (ISRA), Thiès, Senegal; ^4^ Oil Crops Research Program, National Semi-Arid Resources Research Institute (NaSARRI), Soroti, Uganda; ^5^ School of Plant and Environmental Sciences, Tidewater Agricultural Research and Extension Center (AREC), Virginia Tech, Suffolk, VA, United States; ^6^ Texas A&M AgriLife Research and Department of Plant and Soil Science, Texas Tech University, Lubbock, TX, United States; ^7^ Institute of Plant Breeding Genetics and Genomics, University of Georgia, Tifton, GA, United States

**Keywords:** candidate genes, environmentally friendly, genomics, markerassisted selection, oilseed, early leaf spot, late leaf spot

## Abstract

Early leaf spot (ELS) and late leaf spot (LLS) diseases are the two most destructive groundnut diseases in Ghana resulting in ≤ 70% yield losses which is controlled largely by chemical method. To develop leaf spot resistant varieties, the present study was undertaken to identify single nucleotide polymorphism (SNP) markers and putative candidate genes underlying both ELS and LLS. In this study, six multi-locus models of genome-wide association study were conducted with the best linear unbiased predictor obtained from 294 African groundnut germplasm screened for ELS and LLS as well as image-based indices of leaf spot diseases severity in 2020 and 2021 and 8,772 high-quality SNPs from a 48 K SNP array Axiom platform. Ninety-seven SNPs associated with ELS, LLS and five image-based indices across the chromosomes in the 2 two sub-genomes. From these, twenty-nine unique SNPs were detected by at least two models for one or more traits across 16 chromosomes with explained phenotypic variation ranging from 0.01 - 62.76%, with exception of chromosome (Chr) 08 (Chr08), Chr10, Chr11, and Chr19. Seventeen potential candidate genes were predicted at ± 300 kbp of the stable/prominent SNP positions (12 and 5, down- and upstream, respectively). The results from this study provide a basis for understanding the genetic architecture of ELS and LLS diseases in African groundnut germplasm, and the associated SNPs and predicted candidate genes would be valuable for breeding leaf spot diseases resistant varieties upon further validation.

## Introduction

Groundnut [*Arachis hypogaea* L. (2n = 4x = 40)] is an allotetraploid originating from South America and is a grain legume that is largely grown in the tropical and subtropical regions of the world (Bertioli et al., 2016). It comprises of two genomes with their origins coming from different diploid wild ancestors (Bertioli et al., 2016). Groundnut plays a pivotal role in the life of small-holder farmers in Ghana, and it is a suitable vehicle for making improvements in the areas such as poverty alleviation, food and nutritional security (Tyroler, 2018). It is a good source of plant protein for resource poor-households who are unable to buy animal protein. Groundnut also provides vitamins, minerals and unsaturated oil for most Ghanaians (Asibuo et al., 2008). Increase production and consumption of groundnut will reduce the number of the over 800 million people in many developing countries who are chronically hungry as well as the about 2 billion people who suffer micronutrients deficiencies (FAO et al., 2019). Globally, it provided 8, 3 and 2% of oilseed produced, vegetable oil and protein meal consumed, respectively (http://soystats.com/, accessed 03.07.2022).

Despite the importance of groundnut, its cultivation is hindered by numerous biotic and abiotic factors. Early leaf spot (ELS) caused by the fungus *Passalora arachidicola* (S. Hori) and late leaf spot (LLS) also caused by the fungus *Nothopassalora personata* (Berk. & Curt.) are the two most destructive groundnut diseases in Ghana with a potential yield loss of ≤ 70% (Naab et al., 2005; Denwar et al., 2021). Chemical control of these diseases is not feasible in farmers field in Ghana due to their inability to afford these chemicals and therefore ends up not controlling the diseases (Nutsugah et al., 2007; Denwar et al., 2021). Farmers often confuse leaf spots severities with maturity indicator further affecting their mitigation measures. Development and cultivation of leaf spot resistant varieties is cheaper and environmentally friendly (Gaikpa et al., 2017). Resistance is largely controlled by polygenes and influenced by genotype (G), environment (E) and their interactions (G×E) (Johnson, 1984; Wiesner-Hanks and Nelson, 2016). This makes the traditionally commonly used screening methods for identifying leaf spot resistant varieties in Ghana difficult due to the partial and polygenic nature of these diseases (Dwivedi et al., 2002; Pasupuleti et al., 2013). In addition, conventional phenotyping procedures are laborious, time-consuming, destructive, subjective, costly, inefficient and lack inter or intra-rater repeatability (Araus et al., 2018; Awada et al., 2018).

To overcome errors and expenses by manual phenotyping, the red-green-blue (RGB)-image method (which is the science of making measurements through the use of an RGB camera), together with conventional and marker-assisted selection (MAS) may overcome the flaws of current breeding methods (Li et al., 2014; Wang et al., 2017; Yang et al., 2020; Sarkar et al., 2021a). The application of the RGB image method for screening has generated much interest in agricultural research because of its importance in crop production (Sie et al., 2022; Chapu et al., 2022). RGB image method is more efficient, offers inter or intra-rater repeatability, is easy to apply, is less expensive, non-destructive, and offers the chance to take multiple measurements on a specific plant due to the non-destructive nature of the technology (Araus et al., 2018; Awada et al., 2018; Gill et al., 2022). Moreover, the application of the RGB image method in phenotyping will allow the screening of a large set of genotypes using a small fraction of the time that would have been used in conventional phenotyping. For instance, previous studies have shown the efficacy of the RGB imaging for assessment of a number of diseases in several crops verticillium wilt (caused by *Verticillium dahliae* Kleb) in olive (*Olea europaea L.*) (Sancho-Adamson et al., 2019), yellow rust (*Puccinia striiformis* f. sp tritici) in wheat (Zaman-Allah et al., 2015; Zhou et al., 2015), and lethal necrosis (caused by a combination of maize chlorotic mottle virus (MCMV) and sugar cane mosaic virus (SCMV) in maize (*Zea mays* L.)) (Kefauver et al., 2015).

In the last decade, there has been rapid development of next-generation sequencing, high-throughput genotype data together with phenotypic data for utilization to identify marker-trait associations *via* genome-wide association study (GWAS) (Varshney et al., 2019; Varshney et al., 2020). Compared to linkage mapping, GWAS has emerged as a powerful tool to detect markers (single nucleotide polymorphisms (SNPs)) closely linked to quantitative trait loci (QTL), based on the principle of linkage disequilibrium (LD) between genetic markers and QTL that affect the trait (Geng et al., 2015). By this strategy, Zhang et al. (2020a) detected a total of 46 and 28 QTL for ELS and LLS, respectively, with Efficient Mixed-Model Association eXpedited (EMMAX), while Pandey et al. (2014) detected 6 QTL for ELS and 1 QTL for LLS. In contrast, seven and five major QTL for ELS and LLS have been detected and reported on chromosomes 2 and 3, respectively (Chu et al., 2019). In addition, Shaibu et al. (2021) detected 25 SNPs for ELS in mini-core collection of 168 accessions in Nigeria by efficient mixed-model association (EMMA) and compression mixed linear model (CMLM).

The statistical model adopted is one of the setbacks to the power of detection in GWAS (Gupta et al., 2014; Ibrahim et al., 2020; Yoosefzadeh-Najafabadi et al., 2022). Traditional popular statistical models (single-marker genome-wide scan models), mixed linear model (MLM), and general linear model (GLM), among others, have a number of limitations such as the stringent threshold of significance and mapping power (Wen et al., 2018). To overcome these limitations, several multi-locus models have been developed and utilized for GWAS in several crops (Zhang et al., 2019; Karikari et al., 2020; Berhe et al., 2021; Vikas et al., 2022). Among them include a multi-locus random-SNP-effect mixed linear model (mrMLM) (Wang et al., 2016), a fast mrMLM (FASTmrMLM) (Zhang et al., 2018), a fast mrMLM efficient mixed-model association (FASTmrEMMA) (Wen et al., 2018), polygene-background-control-based least-angle regression plus empirical Bayes (pLARmEB) (Zhang et al., 2017), Kruskal-Wallis test with empirical Bayes under polygenic background control (pKWmEB) (Ren et al., 2018) and integrative sure independence screening expectation maximization Bayesian least absolute shrinkage and selection operator model (ISIS EM-BLASSO) (Tamba et al., 2017).

The multi-locus models have become the state-of-the-art procedure to identify genetic bases for complex traits due to their power of detection and robustness (Zhang et al., 2019). Therefore, the present study applied the six multi-locus models to identify genomic regions and potential candidates associated with ELS and LLS diseases. A total of 294 groundnut accessions were collected from different African countries and screened in two years with manual scoring of ELS and LLS together with 5 imaged-based indices and area under disease progression curve (AUDPC) for both diseases. The results from this study lay the foundation for MAS to speed up breeding for leaf spot-resistant cultivars.

## Materials and methods

### Planting materials and experimental condition

Two hundred and ninety-four African groundnut collections (**Supplementary Table S1**) were planted during the main planting season, from June 2020 to September 2020 and June 2021 to September 2021 at the experimental site (09° 25′ 41″ N, 00° 58′ 42″ W) of Council for Scientific and Industrial Research-Savannah Agricultural Research Institute (CSIR-SARI) located in Nyankpala, Northern region, Ghana. This population comprised 54, 49, 44, 32, 31, 27, 22, 18, and 17 accessions from Uganda, Ghana, Niger, Malawi, Senegal, Mali, Mozambique, Togo, and Zambia, respectively (**Supplementary Table S1**), mainly from African Groundnut Germplasm Collection leaf spots resistant and yield phenotyping programs. This panel was selected for the current study to lay foundation for future molecular breeding. The experimental area is characterized by a relatively dry climate with unimodal rainfall ranging between 500 and 1200 mm annually (Atiah et al., 2019; Atiah et al., 2020). The inception of the rains is in May and ends in October with small scattered precipitations in November. The soils of the research area belong to Ferric Luvisols of the Tingoli series with a brown color, moderately drained, and free from concretions (Atakora and Kwakye, 2016). The experiment was carried out in a location that is a hotspot for the disease and therefore can sufficiently discriminate between susceptible and resistant lines (Danful et al., 2019; Oteng-Frimpong et al., 2021)

The accessions were arranged in lattice design with three replicates. A plot was made up of one row of 2 m long with a spacing of 0.5 m between rows and 0.2 m between plants. One seed was planted per hill. Weeding was carried out whenever necessary to ensure a weed-free trial.

### Agronomic practices

Pre-emergence weed control was done by spraying (Alligator^®^ 400EC, Pendimethaline 400g/L, EC) and glyphosate (480g/L SL) at 200 ml/15 liters of water immediately after planting. Weeds were manually controlled regularly by hoeing between the rows and pulling weeds within rows as well as on top of plots using hands to ensure a weed-free experiment. Earthen-up was done 40 days after planting to enhance aeration. A compound fertilizer made of nitrogen(N), phosphorus (P), potassium (K) together with sulphur (S), zinc (Zn) and boron (B), i.e., (N:P:K: 11:22:21+5S+0.7Zn+0.5B) was applied on the sides of the plants two weeks after seedling emergence at a rate of 150 kg/ha. At the same time, the experiment was sprayed against aphis infestation using *K*-Optimal (Lambda-cyhalothrin 15 g/l + acetamiprid 20 g/l; EC) at 40 ml in a 15L Knapsack sprayer.

### Collection of phenotypic data

#### Visual scoring for leaf spot disease

Visual scoring for the severity of ELS and LLS infection was evaluated using the scale described by Subrahmanyam et al. (1995) at 70 and 90 days after planting (Sie et al., 2022). Values of 1 to 4 indicate increasing leaf spot incidence on leaflets within the lower or upper canopy, but no defoliation. Ratings from 4 to 10 are associated with increasing levels of severity with defoliation (Chiteka et al., 1988). The average score of the two-sampling time was computed. AUDPC values were computed for each plot from these disease ratings using the formula: $\text{AUDPC}=\sum_{i=1}^{\text{a}}\left[\left\{\frac{{\text{y}}_{\text{i}}+{\text{y}}_{\text{i}+1}}{2}\right\}{\text{x(t}}_{\text{i}+1}-{\text{t}}_{\text{i}})\right]$ , where y_i_ is the level of disease severity score at a point in time, _t(i+1)_-t_i_ is the number of days between two successive scores (Shaner and Finney, 1977).

#### Measurement of normalized difference vegetation index

GreenSeeker^®^ handheld sensor: (Model HCS-100 manufactured by Trimble Navigation Limited, Sunnyvale, USA) was used to measure the canopy normalized difference vegetation index **(**NDVI) of the vegetation from each plot. The instrument was aligned horizontally and maintained at a constant height of 50 cm over the plants’ canopy with a walking speed to cover the row within 60 seconds. The GreenSeeker optical sensor uses radiation of 650 ± 10 of red and 770 ± 15 of near-infrared band independently. The sensor uses built-in software to directly calculate the NDVI value using the formula: (NIR-RED)/(NIR+RED) (Rouse et al., 1974). The NDVI value which ranges from 0.00 to 0.99 was recorded from the screen of the device. Readings were taken at 70 and 90 days after planting when the sun was at its zenith.

#### RGB images

The RGB digital camera (Samsung Galaxy NX300) was used to take close-up images of one plot at a time. The camera was set to “auto” to allow the camera to adjust the required sharpness, brightness, and hue depending on the light available, with the zoom of the lens being at 0. A part representing the plot was selected for the image. The camera was maintained at the same height of 80 cm over the row for all pictures and facing the sun to avoid any shadows on the pictures. Pictures were taken at 70, and 90 days after planting. Digital image analysis was carried out in Image J software by converting hue (H), saturation (S), and brightness (B) values into the dark green color index (DGCI).

#### Green area, greener area, and crop senescence index

Green area (GA=H 60-120°), greener area (GGA= H 80-120°), Hue angle, and crop senescence index (CSI=(100*(GA-GGA)/GA) (Gracia-Romero et al., 2018) were extracted using Breedpix 2.0 option from the CIMMYT maize scanner 1.16 plugin (http://github.com/george-haddad/CIMMYT open software; Copyright 2015 Shawn Carlisle Kefauver, University of Barcelona); produced as part of Image J/Fiji (open source software; http://fiji.sc/Fiji) (Schindelin et al., 2012; Schindelin et al., 2015). Both GA and GGA measure the number of green pixels on an image. However, the GGA removes green tones that are yellowish from the image and, accordingly, differentiates leaf senescence and active photosynthetic biomass more accurately.

### Statistical analysis of phenotypic data

Data collected manually and imaged based across the two years (2020 and 2021) were subjected to analysis of variance (ANOVA) in SAS (SAS Institute, 2010. SAS/STAT software version 9.2. SAS Institute Inc, Cary, NC) with a general linear model procedure (PROC GLM), following statistical model y_pqr_=μ+G_p_+E_q_+GE_pq _+R_r(q)_+ϵ_pqr_ , where y_pqr_ stands for the individual observation of pqr^th^ experiment unit, μ is the total average phenotypic value, G_p_ is the effect of the p^th^ genotype, E_1_is the effect of the q^th^ year, GE_pq_ is the interaction effect between the p^th^ genotype and the q^th^ year, R_r(q)_ is the effect of the r^th^ block within the q^th^ year, and ε_pqr_ is the residual error. All factors were considered random.

Descriptive statistics: mean, standard error of the mean, kurtosis, and skewness were calculated in SAS (SAS Institute, 2010. SAS/STAT software version 9.2. SAS Institute Inc, Cary, NC) from the two years data. Pearson correlation coefficients were computed and visualized in R with the corrplot package (Wei et al., 2017).

In addition to the above, broad-sense heritability (H^2^) for each trait was computed following the formula proposed by Nyquist and Baker (1991), thus ${\text{H}}^{2}=\sigma_{\text{g}}^{2}\;/(\sigma_{\text{g}}^{2}+{\text{σ}}_{\text{ge}}^{2}/\text{n }+\sigma_{\text{e}}^{2}/\text{nr})$ where $\sigma_{\text{g}}^{2}$ is the genotypic variance, ${\text{σ}}_{\text{ge}}^{2}\;$ is the genotype by environment interaction variance, $\sigma_{\text{e}}^{2}\;$ is the error variance, n is the number of environments, and r is the number of replications.

### Genotyping and population structure analysis

Prior to genotyping, fresh and healthy leaf samples were collected from the panel evaluated in this study and stored at -80 °C for DNA isolation. The genomic DNA was extracted using the modified CTAB method (Porebski et al., 1997). Purified DNA was dissolved in TE buffer for further analysis. The quantity and quality of the DNA were assessed with NanoDrop™ 2000 Spectrophotometer (Thermo Scientific, Wilmington, DC, USA). The genotyping was performed using SNP array (Affymetrix 2). The SNP array used in this study was the 48 K SNP array that was developed for Arachis Axiom Arachis. Quality control was conducted following the procedure outlined by Clevenger et al. (2018) on 8,911 SNPs.

Population structure was analyzed *via* Structure software 2.3.3 (Pritchard et al., 2000) with the number of presumed population (K) set from 1-7 and replicated 5 times with a burn-in period of 50,000 steps and Monte Carlo Marko Chain of 100,000. An admixture model with correlated allele frequency was adopted in this study (Falush et al., 2003). After analysis, the Structure Harvester online program (https://taylor0.biology.ucla.edu/structureHarvester/) was used to retrieve the optimum K (ΔK) (Earl and Vonholdt, 2012). Only the accessions with a membership coefficient (Q) ≥ 0.60 were assigned to a genetic group and those with Q<0.60 were classified as admixture (Delfini et al., 2021). We further constructed a neighbor-joining (NJ) phylogenetic tree *via* TASSEL 5.2.31 software (Bradbury et al., 2007). A kinship plot was produced with the kinship2 package in R (Sinnwell et al., 2014). LD between pairwise SNPs was computed with RTM-GWAS V1.1 software with squared allele frequency correlation model (He et al., 2017). The panel’s LD decay rate was estimated as the chromosomal distance when the LD decay (r^2^) fell to half of its highest value. The graph of the LD decay was produced with the help of GraphPad Prism version 5.01 (GraphPad Software, San Diego California USA) within the pairwise distance of 5 Mb in the genome.

### Marker-trait association analysis

To prevent environmental (year) variation in phenotypic data, the best linear unbiased predictor (BLUPs) for each accession for all traits were calculated using R package lme4 (Bates et al., 2015) with the effect of environment (year), replicate within E, G, GE and error as random effects. Six multi-locus models, i.e., mrMLM (Wang et al., 2016), FASTmrMLM (Zhang et al., 2018), FASTmrEMMA (Wen et al., 2018), pLARmEB (Zhang et al., 2017), pKWmEB (Ren et al., 2018) and ISIS EM-BLASSO (Tamba et al., 2017) were conducted in R with mrMLM package (V4.0.2) (Zhang et al., 2020b) with both Q matrix and principal component (PC) together with kinship matrix. The threshold with a critical logarithm of odd value was set at 3.

### Candidate gene prediction and *in silico* analyses

SNPs detected for at least two traits were considered stable, hence were selected for downstream analysis including candidate gene prediction from reference genome (*A. hypogaea* V1) (Bertioli et al., 2019) available on phytozome (https://phytozome-next.jgi.doe.gov/). The gff3 file was retrieved from the phytozome website (https://phytozome-next.jgi.doe.gov/) and information gene ontology (GO) (Ashburner et al., 2000), protein families (Pfam) (Bateman et al., 2004), Kyoto Encyclopedia of Genes and Genomes (KEGG) (Kanehisa and Goto, 2000), and transcription factors (Jin et al., 2014)) were further explored in selecting candidate genes with the SNP ± LD.

## Results

### Phenotypic variation, broad-sense heritability, and correlation among the 294 accessions of groundnut

**Figure 1:**
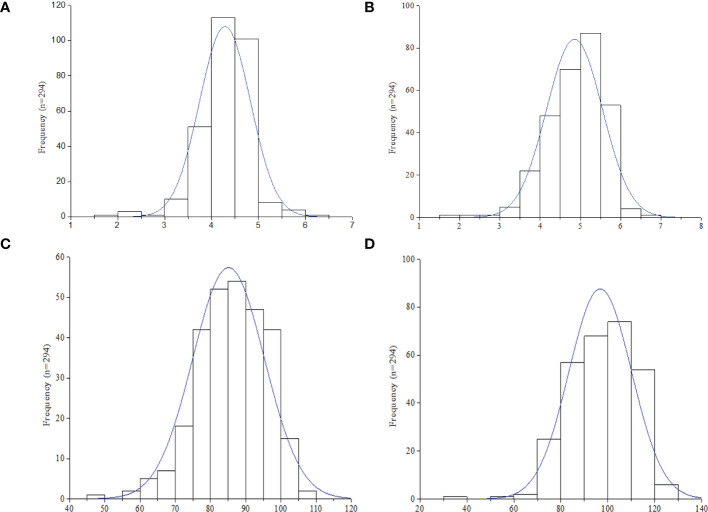
Frequency distribution of leaf spot diseases rating in the 294 accessions (n) in this study. **(A)** Early leaf spot (ELS). **(B)** Late leaf spot (LLS). **(C)** Area under disease progression curve (AUDPC) -ELS. **(D)** AUDPC - LLS.

Descriptive statistics and H^2^ of the four image-based traits (Hue, GA, GGA, and CSI), vegetative index trait (NDVI), manually scored traits (ELS and LLS), and quantitatively computed traits (AUDPC-ELS and -LLS) among the 294 groundnut accessions based on the two years (2020 and 2021) evaluation in this study are shown in Table. The hue, GA, GGA, CSI (from the image-based phenotyping) and NDVI values ranged (mean ± Standard error of mean) from 38.37-90.20 (68.03 ± 0.56), 0.30-0.92 (0.63 ± 0.01), 0.23-0.81 (0.48 ± 0.01), 9.27-45.90 (26.19 ± 0.45) and 0.36-7.09 (0.68 ± 0.02), respectively (**Table 1**). The qualitatively scoring of ELS and LLS incidence followed a normal distribution (**Figures 1A, B**), with the mean scores of 4.28 ± 0.03 and 4.85 ± 0.04 (**Table 1**), respectively. This indicates that the population used for this study exhibited a wide range of variation in response to ELS and LLS diseases. Also, the quantitatively computed AUDPC for ELS and LLS ranged from 30.00-140.00 (86.00 ± 0.60) and 40.00-140.00 (97.00 ± 0.47) (**Table 1**), respectively, and this followed normal distribution among the population used in this study (**Figures 1C, D**).

**Table 1:** Descriptive statistics and broad-sense heritability of nine traits.

Parameter ^a^	Mean ± SEM ^b^	Range	CV (%) ^c^	Skewness	Kurtosis	H^2^ (%) ^d^
Hue*	68.03 ± 0.56	38.37-90.20	13.85	0.16	-0.26	90.09
GA*	0.63 ± 0.01	0.30-0.92	17.96	0.34	-0.48	90.68
GGA*	0.48 ± 0.01	0.23-0.81	25.39	0.54	-0.85	92.38
CSI*	26.19 ± 0.45	9.27-45.90	29.68	-0.12	-0.99	82.12
NDVI*	0.68 ± 0.02	0.36-7.09	55.69	16.42	276.80	66.23
ELS^+^	4.28 ± 0.03	1.58-6.17	12.66	-0.88	4.01	73.08
LLS^+^	4.85 ± 0.04	1.58-6.67	14.33	-0.85	1.56	85.55
AUDPC-ELS	86.00 ± 0.60	30.00-140.00	28.53	0.05	-0.93	75.97
AUDPC-LLS	97.00 ± 0.47	40.00-140.00	19.87	-0.13	-0.54	86.00

^a^Hue, hue angle; GA, green area; GGA, greener area; CSI, crop senescence index; NDVI, normalized difference vegetation index; ELS, early leaf spot; LLS, late leaf spot; AUDPC-ELS and -LLS, area under disease progress curve for ELS and LLS, respectively. * RGB-image-based phenotyping.+ Manual disease scoring with a scale of 1-9 by Subrahmanyam et al. (1995). ^b^mean± standard error of mean. ^c^Coefficient of variation. ^d^Broad-sense heritability.

Combined ANOVA of the two years data revealed that eight traits varied significantly due to genotypes (G), environment (E), and their interaction (GE), while NDVI differed due to E (**Supplementary Tables 2A–I**). These coupled with high H^2^ (**Table 1**) suggest that these ELS and LLS are largely controlled by polygenes with both major and minor effects. This highlights that selection based only on phenotypic variation may be misleading.

We further conducted a Pearson correlation analysis among the five image-based indices, ELS, LLS, AUDPC-ELS, and -LLS with Corrplot package in R (Wei et al., 2017) and the results are shown in **Supplementary Figure 1**. Among the five image-based indices, only CSI positively correlated with LLS (correlation coefficient, r = 0.71), AUDPC-LLS (r=0.66), ELS (r=0.56) and AUDPC-ELS (r=0.44) (**Supplementary Figure 1**). This indicates that CSI could be a relatively good indicator for the assessment of ELS and LLS diseases. However, the qualitatively and quantitatively scoring of ELS and LLS negatively correlated with the other four image-based indices (GA, GGA, Hue, and NDVI) (r=-0.02 to -0.82) (**Supplementary Figure 1**) confirming that leaf spot diseases affect the photosynthetic capacity of the leaf. These together with leaf spot diseases ratings and ANOVA highlight that the phenotypic data from the population qualify for GWAS mapping.

### Genetic differentiation and LD estimation among the mapping population

After quality control on the obtained 8,911 SNPs, a total of 8,772 SNP markers were distributed between the two sub-genomes (A and B) with quality criteria of minor allele frequency< 0.05 and call rate<0.95. Out of these, 8,152 SNPs were located on one of the linkage groups in the two sub-genomes (A and B), while 619 SNPs were located on scaffolds. The SNPs located on the scaffolds were excluded from the downstream analysis. The longest and shortest chromosome (Chr) spanned 149.79 Mb (Chr15) and 49.33 Mb (Chr08), respectively (**Table 2**; **Supplementary Figure 2**). However, Chr08 and Chr19 contained the highest and least number of SNPs (877 and 191, respectively).

**Table 2:** Distribution of single nucleotide polymorphism (SNP) markers between A and B sub-genomes of groundnut (chromosome) of the studied population.

Genome/LG	Chr.	Length (bp)	Length (kb)	Length (Mb)	No. of SNPs	Kbs/SNP	SNPs/Mb
A01	1	106806005	106806.01	106.81	444	240.6	4.16
A02	2	93528862	93528.86	93.53	327	286.0	3.50
A03	3	134894015	134894.02	134.89	406	332.3	3.01
A04	4	121312866	121312.87	121.31	429	282.8	3.54
A05	5	109393004	109393.00	109.39	393	278.4	3.59
A06	6	112315382	112315.38	112.32	428	262.4	3.81
A07	7	78545074	78545.07	78.55	312	251.7	3.97
A08	8	49330572	49330.57	49.33	191	258.3	3.87
A09	9	120497462	120497.46	120.50	267	451.3	2.22
A10	10	109302486	109302.49	109.30	230	475.2	2.10
B01	11	137285820	137285.82	137.29	325	422.4	2.37
B02	12	108946667	108946.67	108.95	247	441.1	2.27
B03	13	135317267	135317.27	135.32	325	416.4	2.40
B04	14	132798623	132798.62	132.80	365	363.8	2.75
B05	15	149794401	149794.40	149.79	334	448.5	2.23
B06	16	136159078	136159.08	136.16	433	314.5	3.18
B07	17	126126609	126126.61	126.13	507	248.8	4.02
B08	18	129540280	129540.28	129.54	610	212.4	4.71
B09	19	147063990	147063.99	147.06	877	167.7	5.96
B10	20	135921470	135921.47	135.92	702	193.6	5.16
**Total**		**2374879933**	**2374879.93**	**2374.88**	**8152**	**6348.00**	**68.82**

LG, Linkage group; Chr, Chromosome.

**Figure 2:**
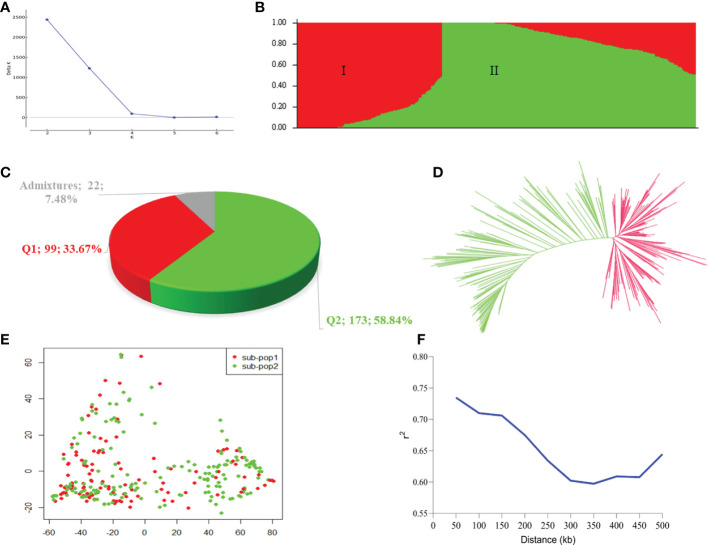
Population stratification of the 294 accessions in this study. **(A)** Estimated ΔK in the population structure analysis retrieved from STRUCTURE HARVESTER online programme. **(B)** Population structure by STRUCTURE software. The two colours (red and green) represent two sub-populations. Each colour represent one inferred ancestral population with the vertical column representing one individual accession and coloured segment in each column denotes percentage of the individual inferred ancestral population in the studied panel. **(C)** Proportion of pure and admixture lines based on membership coefficients (Q) ≥0.60 as pure lines, while those with Q< 0.60 considered as admixture lines. **(D)** A neigbour-joining phylogenetic tress. **(E)** Principal component analysis plot. **(F)** Linkage disequilibrium decay plot within the 500 kb across the two sub-genomes **(A, B)**.

The Bayesian model based population structure analysis was carried out with K=1-7 with 5 independent runs for each K. The estimated ΔK plot shown in **Figure 2A** reveals that 294 accessions are optimally grouped into two sub-populations (I and II) (**Figure 2B**). The sub-population I consisted of 99 accessions (≈33.67%) with nearly 50% of them from Uganda and Senegal, while those in sub-population II comprised 173 accessions (≈58.84%) with 43, 26, 20, and 20 accessions from Niger, Ghana, Malawi, and Mali, respectively (**Table 3**; **Supplementary Table 1**). Based on the Q≥ 0.60 as pure lines, twenty-two accessions representing 7.48% of the population are considered admixtures (**Figure 2C**). Allele frequency divergence between two sub-populations was estimated as 0.38, while the expected heterozygosity between individual accessions with sub-population I and II are 0.15 and 0.18, indicating that sub-population II is relatively more diverse than sub-population I. The population structure stratification was consistent with kinship matrix, phylogenetic trees, and principal component analysis (**Figures 2D, E**). The LD decay across the two sub-genomes of the studied panel was estimated to be about 300 kbp (**Figure 2F**).

### Marker-trait association

**Figure 3:**
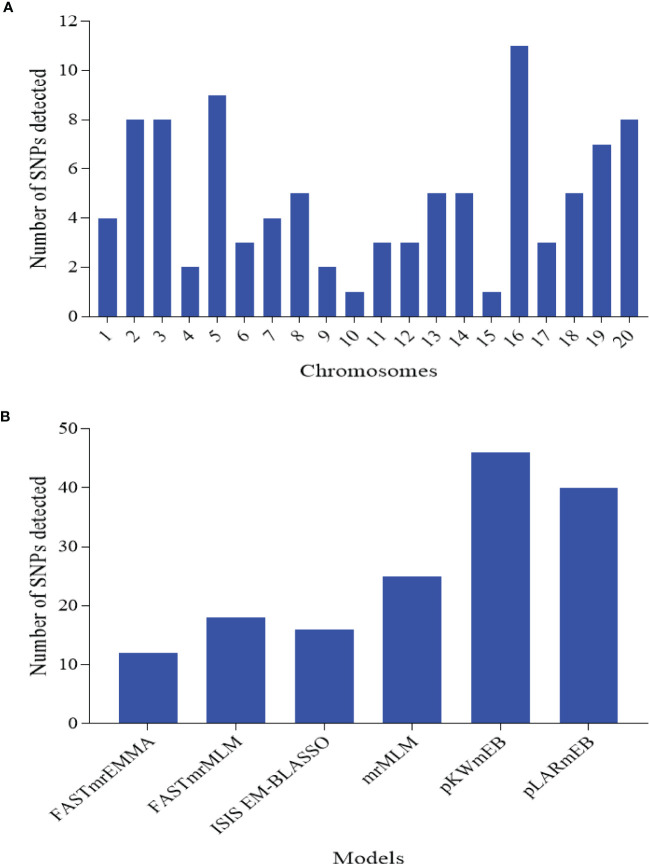
Number of significantly associated single nucleotide polymorphism (SNP) markers detected for the nine traits associated with leaf spot diseases rating. **(A)** SNPs detected on each chromosome of the two sub-genomes. A sub-genome comprised chromosome (Chr) 1 to 10, while B sub-genome consisted of Chr11-20. **(B)** SNPs detected by each of the six multi-locus models implemented in this study.

In order to remove environmental effect during the marker-trait association mapping, BLUP values were used where the effect of environment (year), replicates within E, G, GE, and error were considered as random effects. With the six multi-locus models, a total of 97 SNPs distributed across the 20 chromosomes with an average of ≈5 SNPs per Chr and range of 1 SNP on Chr15 to 11 SNPs on Chr16 were detected (**Figure 3A**; **Supplementary Table 3**). Of these, the power of detection among the six models followed pKWmEB (46 SNPs) > pLARmEB (40 SNPs) > mrMLM (25 SNPs) > FASTmrMLM (18 SNPs) > ISIS EM-BLASSO (16 SNPs) > FASTmrEMMA (12 SNPs) (**Figure 3B**).

Comparative analysis among the six multi-locus models revealed that twenty-nine unique SNPs were detected by at least two models for one or more traits across 16 chromosomes with exception of Chr08, Chr10, Chr11 and Chr19 (LOD ≥ 3.00) (**Table 4**). Among these, seven SNPs (AX-176823205 (Ch01), AX-176823123 (Chr01), AX176799357 (Chr02), AX-176796174 (Chr05), AX-147224865 (Chr06), AX-147239793 (Chr06) and AX-177643984 (Chr20)) associated with two traits (**Table 4**). For example, SNP, AX-176823205 associated with both Hue and LLS with allele C at 91414269, LOD of 3.40-5.04 and phenotypic variation explained (PVE) of ≤ 3.12%. The C allele had negative and positive effect on Hue and LLS, respectively. This and the other six SNPs (AX-176823123, AX176799357, AX-176796174, AX-147224865, AX-147239793 and AX-177643984) may be the basis for the r values observed on **Supplementary Figure 1**. Therefore, these SNPs could be valuable genetic resources to understand the relationship among the evaluated indices associated with leaf spot diseases.

In addition, nine SNPs (AX-176799357, AX-176806210, AX-176796174, AX-176793720, AX-147224496, AX-176818776, AX-176820950, AX-177643984 and AX-133120520) were associated with at least one model with PVE ≥ 10%, hence these were considered as major SNPs for downstream analysis for candidate genes prediction (**Table 4**). Interestingly, AX-176799357 was linked to both LLS and AUDPC-LLS, AX-176796174 associated with both Hue and AUDPC-ELS, and AX-133120520 associated with both GA and GGA.

As typical of quantitative traits, seventy SNPs were trait and model specific with LOD and PVE ranging 3.04 (AX-177637712 on Chr17 with LLS) to 35.90 (AX-147227883 on Chr07 with GGA), and<0.01% (AX-177637712 on Chr17 with LLS) to 21.79% (AX-176797562 on Chr02 with CSI) (**Supplementary Table 3**). These markers may need further verification for their possible use in practical plant breeding.

### Candidate gene prediction around stable SNPs

To predict candidate genes, significant SNPs with at least 1 of the following criteria: linked to more than 1 trait, detected by at least 4 GWAS model and with PVE ≥ 10% were used for gene mining. Two hundred and fifty-three genes located within 300 kbp of 14 SNPs were mined and *in silico* analysis performed. In all, 17 potential candidate genes were predicted, of which 12 and 5 are located down- and up-stream of the linked SNP positions, respectively (**Table 5**). BAEJ4E gene is located 172.3 kbp up-stream of AX-176823205 (chr01) was predicted for Hue and LLS traits, and this gene encodes for glutathione S-transferase which have been reported to regulate plant response to fungal infection (Dean et al., 2005; Gullner et al., 2018). In addition, four genes (7U0T6N, CA7A2G, YE4BG5 and AZU29N predicted for CSI & ELS, LLS & AUDPC-LLS, AUDPC-LLS, and GA & GGA, respectively) are involved in photosynthesis pathway, hence could be involved in regulating groundnut response to leaf spot pathogens. Phytohormones are reported to regulate plant’s response to disease attack or resistance (Denancé et al., 2013). 5L52L8 gene is involved in ethylene biosynthesis, and L76SLB gene for auxin efflux carrier. Both of genes (5L52L8 and L76SLB) were located ≤ 289 kbp from the AX-176799357 on Chr02 linked to LLS and AUDPC-LLS, and AX-176793720 on Chr05 linked AUDPC-LLS, respectively (**Table 5**).

**Figure 4:**
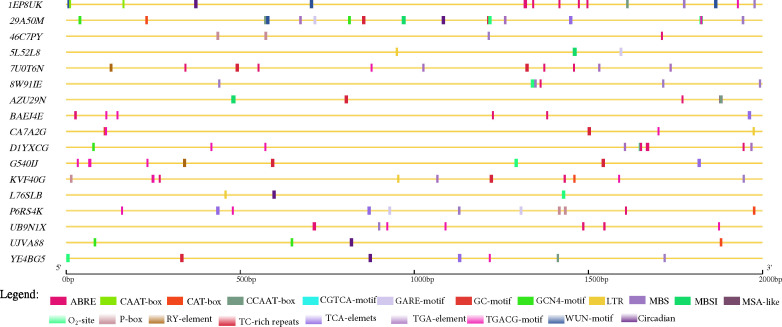
Key cis-acting regulatory elements (CAREs) identified in the promoter regions (2 kbp upstream) of the 17 predicted candidate genes in this study retrieved from PlantCare database (Lescot et al., 2002). ABRE= abscisic acid responsiveness; CAAT-box= essential CARE, CAT-box= CARE related to meristem expression, CCAAT-box= MYBHv1 binding site, GARE-motif= gibberellin-responsive element, GC-motif= anoxic specific inducibility, GCN4-motif= involved in endosperm expression, LTR= low temperature responsiveness, MBS= MYB binding site involved in drought-inducibility, MBSI= MYB binding site involved in flavonoid biosynthetic genes regulation, MSA-like= cell cycle regulation, O_2_-site= zein metabolism regulation, P-box= gibberellin-responsive element, RY-element= seed-specific regulation, TC-rich repeats= defense and stress responsiveness, TCA-elements= salicylic acid responsiveness, TGA-element= auxin-responsive element, TGACG-motif= MeJA-responsiveness, and, WUN-motif= and Circadian= circadian control.

We further explored the promoter region (2 kbp) of the 17 candidate genes predicted for Cis-acting regulatory elements (CAREs) with PlantCare database (http://bioinformatics.psb.ugent.be/webtools/plantcare/html (Lescot et al., 2002) that may be involved in the modulating groundnut response to leaf spot diseases. Aside essential CAREs (CAAT- and TATA-box) as well as 20 light responsiveness CAREs (such as AE-box, AT1-motif, Box 4, G-Box, GATA-motif, etc.), twenty CAREs with potential in modulating gene expression were identified (**Supplementary Table 5**; **Figure 4**). Seven genes (29A50M, 7U0T6N, AZU29N, CA7A2G, G540IJ, KVF40G, and YE4BG5) were found to contain at least one ATTCTCTAAC (TC-rich repeats) demonstrated to involve in defence and stress responsiveness in Nicotiana tabacum (**Figure 4**) (Diaz-De-Leon et al., 1993; Xu et al., 2011; Wang et al., 2020). Moreover, the role of salicylic acid (SA) in plant defence is well documented, thus SA is required for basal resistance against pathogens as well as for the inducible defence mechanism, systemic acquired resistance which confers resistance against a broad-spectrum of pathogens including *P. arachidicola* and *N. personata* (Chaturvedi and Shah, 2007). Five predicted candidate genes (29A50M, BAEJ4E, G540IJ, P6RS4K and YE4BG5) (**Table 4**) contain at least one TCA-element (TCAGAAGAGG) involved in SA responsiveness (**Figure 4**).

Other CAREs that may be implicated in groundnut response to leaf spot pathogens identified include (abscisic acid (ABA), gibberellin (GA), auxin, methyl jasmonate (MeJA), MYB binding site involved in flavonoid biosynthetic genes regulation (MBSI) and so) were detected in at least 1 of the predicted candidate genes highlighting the possibility of their involvement in modulating groundnut response to leaf spot pathogen. The actual roles of the 17 predicted candidate genes warrant further screening and functional validation to unravel the bases of correlation among the studied traits (**Supplementary Table S1**).

## Discussion

Leaf spots (ELS and LLS) diseases are the two most destructive groundnut diseases in Ghana resulting in ≤ 70% yield losses which is controlled largely by chemical method (Naab et al., 2005; Denwar et al., 2021). To speed up breeding efforts, the present study was undertaken to identify significantly associated molecular markers and putative candidate genes linked to ELS and LLS diseases’ indicators. Irrespective of the breeding strategy, germplasm serves as lifeblood for breeding effort (Acquaah, 2009; Allier et al., 2020). With this, the present study utilized 294 groundnut germplasm assembled from nine African countries (Ghana, Malawi, Mali, Mozambique, Niger, Senegal, Togo, Uganda and Zambia) (**Supplementary Table 1**) to assess their response to leaf spot diseases in 2020 and 2021. These germplasm exhibited wide range of responses to leaf spot diseases (resistance to susceptibility) (**Table 1**; **Figure 1**) of which a portion was recently published by Sie et al. (2022). This suggest that the germplasm hold a promise for breeding groundnut cultivars resistant to leaf spot diseases as well as other demand driven traits. In addition, the high H^2^ (**Table 1**) suggest that these ELS and LLS as well as other indicators used are largely controlled by polygenes with both major minor effects. This highlights that selection based only on phenotypic variation may be misleading.

One of setbacks in phenotyping large germplasm is the laborious nature coupled with human error among others (Sandhu et al., 2022), which have necessitated a number of phenotyping strategies/platforms which complement the conventional phenotyping. Among these include multispectral imaging (Kobayashi et al., 2001; Chang et al., 2021), RGB imaging (Duan et al., 2018) and others (see Sandhu et al. (2022) for more). The present study manually scored leaf spot incidences and these scores were converted to quantitative scores by AUDPC which were correlated by r=0.47-0.76, giving credence to our scorings (**Supplementary Figure 1**). In addition, we used four RGB imaging indices (Hue, GA, GGA and CSI) and one vegetation index (NDVI) to confirm both the manual scoring of ELS and LLS as well as ELS- and LLS-AUDPC. It was observed that CSI positively correlated with ELS, LLS, ELS-AUDPC and LLS-AUDPC with r=0.44-0.71 (**Supplementary Figure 1**), thus the higher the CSI, the more developed the leaf spot diseases, giving an indication that CSI could be used as a screening criterion for leaf spot diseases in groundnut. The predominance and distribution of leaf spots vary according to regions. In most cases there is dual occurrence but the predominance towards physiological maturities varies. For instance, in Eastern Africa, LLS predominates, whereas in Western and central Africa, ELS predominates. However, in the current study, CSI could not distinguish between ELS and LLS, hence further study is needed to develop a model or phenotyping platform to create the distinction.

Recent advances in plant phenotyping involve the use of unmanned aerial vehicles (UAVs) to collect several images generating large amounts of data. Several studies have reported that UAVs are faster and more effective for phenotyping large populations for traits such as height and drought tolerance in groundnut breeding (Sarkar et al., 2021b; Chapu et al., 2022), hence providing the desired high-throughput. This study therefore lays the foundation for investment in such more advanced equipment in groundnut breeding for selection for resistance to late leaf spot and groundnut rosette disease which are the most important foliar diseases in SSA (Chapu et al., 2022).

Leaf spot diseases are well known to affect photosynthetic capacity of leaves (Singh et al., 2011). Three image-based indices, i.e., GA, GGA, Hue and one vegetation index, i.e., NDVI negatively correlated weakly to strongly with ELS, LLS, ELS-AUDPC and LLS-AUDPC (**Supplementary Figure 1**). However, GA, GGA and Hue seem more better reflective of leaf spot diseases based on our correlation analysis (**Supplementary Figure 1**) According to Wang et al. (2017), NDVI is a proxy of green biomass, which is linked to canopy photosynthesis. Pronounced changes take place in the visible portion of the electromagnetic spectrum due to the effect caused by a disease or physiological stress to the reflectance properties of the vegetation. Healthy plants absorb both the red and blue light of the electromagnetic spectrum, whiles reflecting near-infrared and some part of the green light (Knipling, 1970). Less of the red light is absorbed by a stressed plant as well as reflecting less of the near-infrared light. Wu et al. (2014) have provided evidence that healthy and unhealthy plants differ in their absorption and reflection of visible and near-infrared lights.

The advancement in high-throughput genotyping, next generation sequencing, bioinformatics tools, statistical models, etc., have served as catalyst to access valuable information from genomic databases and a large number of germplasm, allowing effective harnessing of genetic diversity of a crop (Vikas et al., 2022). Such diversity is vital for broadening the genetic base, as it increases the probability of identifying more unique genes for which two parents have different alleles (Mascher et al., 2019). The 294 germplasm optimally divided into 2 sub-populations/clusters/clade (**Figures 2A-E**). This information is not only useful for the utilization of the 294 germplasm, but also provide valuable information in their conservation as the cost of maintenance and uncertainty about their genetic similarity and dissimilarity (Wambugu et al., 2018; Mascher et al., 2019). The results from this study could contribute to tracking the identity of accessions, avoiding unnecessary duplications within and between genebanks and breeding programmes, while maintaining the genetic integrity of accessions (Mascher et al., 2019). The germplasm in sub-populations/clusters/clade did not follow strictly to a country/sub-region (**Table 3**; **Supplementary Table 1**), suggesting that some germplasm may have a duplicated version(s) in other country(ies) breeding programmes.

**Table 3:** Population stratification based on membership coefficient (Q) from the STRUCTURE software.

Country	Number of accessions	Sub-pop I	Sub-pop II	Admixtures
Ghana	49	19	26	4
Malawi	32	10	20	2
Mali	27	4	20	3
Mozambique	22	4	15	3
Niger	44	1	43	0
Senegal	31	19	8	4
Togo	18	6	11	1
Uganda	54	28	22	4
Zambia	17	8	8	1
**Total (%)**	**294**	**99 (37.67%)**	**173 (58.84%)**	**22 (7.48%)**

Accessions assigned to sub-population had Q≥0.60, while those assigned as admixture had Q<0.60.

A number of studies by linkage and association mappings have been conducted on leaf spot diseases in groundnut (Pasupuleti et al., 2013; Pandey et al., 2014; Zhang et al., 2020a; Shaibu et al., 2021). However, most of the reported genomic regions/markers are not consistent due to population specific or environment sensitivity (Patil et al., 2018). This necessitated the present study in our attempt to identify potential markers for marker-assisted selection (MAS) (Barmukh et al., 2022). To increase the chances of detecting more possible SNPs that could be associated with the various indices used to assess leaf spot diseases, we applied 6 multi-locus models (mrMLM, FASTmrMLM, FASTmrEMMA, pLARmEB, pKWmEB and ISIS EM-BLASSO) of GWAS. These models differed in their power of detection of associated SNPs, which are in consonance with several earlier studies (Zhang et al., 2019; Karikari et al., 2020). In comparison to study of Zhang et al. (2020a) who reported only SNPs with major effects (PVE ≥ 10%), the present study detected numerous SNPs with both minor (PVE< 10%) and major effects. According to Zhou et al. (2021) SNPs identified by multiple models are usually reliable when several multi-locus models of GWAS are applied on the same dataset. Hence, AX-176801892 (Chr02, LOD= 3.18-3.99; PVE= 0.05-3.49%) linked to ELS, AX-176799357 (Chr04, LOD= 7.73-16.45; PVE= 12.78-20.69%) linked to LLS, AX-176806210 (Chr04, LOD=3.20-9.84; PVE=7.21-38.50%) linked to AUDPC-LLS, AX-147224496 (Chr04, LOD= 4.36-5.46; PVE= 0.01-10.15%) linked to NDVI and several others (**Table 4**; **Supplementary Table 3**) could be targeted for verification and use for practical plant breeding. The numerous genomic regions/SNPs suggest that leaf spot diseases are regulated by multiple loci with both minor and major effects, hence selection based on only phenotypic data may be misleading (Sood et al., 2020). In addition to the above, a number of SNPs that colocalized with multiple indices assessed in this study could be useful in developing models that could distinguish between ELS and LLS.

**Table 4:** SNPs detected by at least two of the six models for one or more traits.

Trait name ^a^	SNP markers	Chr ^b^	Position (bp)	Models ^c^	LOD ^d^	QTN effect	PVE (%) ^e^	MAF ^f^	Alleles ^g^
Hue	AX-176823205	1	91414269	1,2,3,5	3.40-4.94	-1.09- -1.90	1.49-3.12	0.21	C
LLS				2	5.04	<0.01	0.01		
CSI	AX-176823123	1	96303630	1,2	3.79	0.26-0.47	0.24-1.35	0.22	T
ELS				6	4.01	0.0029	3.02		
AUDPC-ELS	AX-147212206	2	453115	1,5,6	3.68-4.14	0.10-0.20	3.19-7.49	0.14	A
ELS	AX-176801892	2	38320573	1,2,3	3.18-3.99	0.01-0.02	0.05-3.49	0.10	G
LLS	AX-176799357	2	76275147	1,3,6	7.73-16.45	0.09-0.12	12.78-20.69	0.49	T
AUDPC-LLS				4	4.18	1.83	5.16		
ELS	AX-176801892	2	38320573	1,2,3	3.18-3.99	0.01-0.02	0.05-3.49	0.10	G
AUDPC-ELS	AX-147217628	3	118446096	1,2,3,4,6	3.13-7.02	0.14-0.37	5.10-8.51	0.28	G
AUDPC-LLS	AX-176806210	4	27792120	1,2,3,4,5,6	3.20-9.84	3.14-9.91	7.21-38.50	0.13	G
Hue	AX-176796174	5	15450246	1,2,6	4.72-7.31	-3.21- -2.48	10.90-24.54	0.23	T
AUDPC-ELS				5	6.86	0.26	12.22		
Hue	AX-147222698	5	81969778	3,4,5	3.25-7.87	1.15-3.22	3.24-6.16	0.46	G
AUDPC-LLS	AX-176793720	5	102154664	1,2,4,5,6	3.22-5.65	-3.03- -1.83	4.64-11.00	0.35	G
LLS	AX-176808070	6	2451915	1,6	3.09-3.80	0.06	2.34-2.91	0.14	T
NDVI	AX-147224496	6	5557950	3,4,5	4.36-5.46	<0.01	0.01-10.15	0.49	C
GA & GGA	AX-147224865	6	11297985	6	5.47-6.37	<0.01	3.54-3.92	0.12	G
CSI	AX-147228765	7	70634323	1,2,5,6	3.46-5.03	0.92-1.13	1.28-3.74	0.10	T
AUDPC-ELS	AX-147232168	9	1308812	1,2	3.78-3.88	-0.13- -0.09	2.22-4.89	0.32	T
GA & GGA	AX-147239793	12	295929	6	3.88-5.67	<0.01	0.06-2.77	0.50	C
LLS	AX-176804113	13	14456209	1,5,6	4.61-7.46	0.05-0.08	4.03-8.34	0.38	C
CSI	AX-176818776	13	115500602	1,2,6	3.01-3.03	1.21-3.69	12.12-62.76	0.19	C
AUDPC-ELS	AX-147246588	14	2233380	1,2	3.25-3.58	-0.13- -0.08	2.19-5.15	0.45	T
LLS	AX-147247867	14	101149286	1,2,6	3.16-5.88	0.06-0.08	2.56-5.97	0.17	G
AUDPC-ELS	AX-176820950	16	18466678	1,6	5.32-5.41	0.14-0.15	6.84-10.05	0.42	C
CSI	AX-147253729	16	128084428	5,6	3.41-4.21	-0.64- -0.61	3.14-6.33	0.30	C
ELS	AX-177643647	18	7462734	5,6	3.91-6.83	<0.01	0.01-0.58	0.10	G
Hue	AX-147259422	18	127104626	1,2,5	4.21-6.36	-1.95- -1.69	2.03-3.51	0.10	C
Hue	AX-176824170	19	145041727	1,2,5,6	3.08-6.29	-1.84- -1.23	3.03-6.54	0.28	C
AUDPC-ELS	AX-177644589	20	53038882	1,2,6	3.19-4.51	-0.16 - -0.14	5.38-8.79	0.43	C
LLS	AX-177639015	20	121402090	1,5,6	3.96-4.88	0.04-0.06	2.34-5.28	0.43	G
GA & GGA	AX-177643984	20	133120520	6	3.01-3.84	<0.01	2.97-10.44	0.48	G

^a^Hue, hue angle; GA, green area; GGA, greener area; CSI, crop senescence index; NDVI, normalized difference vegetation index; ELS, early leaf spot; LLS, late leaf spot; AUDPC-ELS, area under disease progress curve for early leaf spot; AUDPC-LLS, area under disease progress curve for late leaf spot. ^b^Chromosomes. ^c^mrMLM (1), FASTmrMLM (2), FASTmrEMMA (3), ISIS EM-BLASSO (4), pLARmEB (5) and pKWmEB (6). ^d^Logarithm of odd. ^e^Phenotypic variation explained (%). ^f^Minor allele frequency. ^g^Associated allele. SNP positions, GWAS models and PVE underlined were considered as stable for candidate genes prediction.

One advantage of GWAS is the power to detect SNPs in a narrow genomic regions of which causal variants could result in variation in traits of economic importance such as leaf spot diseases (Jiang et al., 2009; Zhao et al., 2021). However, one of the factors that determine resolution of GWAS is LD (Korte and Farlow, 2013; Ibrahim et al., 2020) which is population specific and influenced by recombination, genetic drift and mating system (Yao et al., 2009). In this study, the LD decay across the two sub-genomes of groundnut was estimated to be about 300 kbp which is nearly 50% higher than the estimation by Zhang et al. (2020a) within 120 kbp in US accessions with 13,382 SNPs. Upon application of LD for the 294 African germplasm, seventeen candidate genes were predicted based on in-silico analyses (**Table 5**; **Figure 4**). A number of these genes encode for phytohormone/contain CAREs/photosynthesis pathway which could be implicated in groundnut response to leaf spot diseases. For plants to defend themselves from pathogen attack, plants often rely on elaborate signaling networks regulated by phytohormones (Kazan and Lyons, 2014). These genes would be valuable for future functional validation by gene overexpression, CRISPR/Cas9 technology, among others (Zaidi et al., 2020).

**Table 5:** Predicted candidate genes around fourteen stable/major SNPs for the studied traits.

	SNP markers			Predicted candidate genes		
Trait name ^a^	Identity ^b^	Chr^c^	Position (bp)	Gene ^d^	Position from SNP (kb)^e^	Domain/function/pathway ^f^
Hue & LLS	AX-176823205	1	91414269	*BAEJ4E*	172.3 (u)	Glutathione S-transferase
CSI & ELS	AX-176823123	1	96303630	*7U0T6N*	299.6 (d)	Oxygenic photosynthesis (pathway), RuBP carboxylase(enzyme)
				*46C7PY*	216.1 (d)	Serine/threonine specific protein phosphatase (Enzyme)
LLS & AUDPC-LLS	AX-176799357	2	76275147	*5L52L8*	247.4 (d)	Ethylene biosynthesis/Superoxide dismutase
				*CA7A2G*	21.4 (d)	Photosystem antenna protein-like
AUDPC-ELS	AX-147217628	3	118446096	*UB9N1X*	111.4 (u)	Ammonium/urea transporter
AUDPC-LLS	AX-176806210	4	27792120	*YE4BG5*	116.7 (d)	Oxygenic photosynthesis (Pathway)
Hue & AUDPC-ELS	AX-176796174	5	15450246	*29A50M*	16.3 (d)	Myc-type, basic helix-loop-helix (bHLH) domain
AUDPC-LLS	AX-176793720	5	102154664	*L76SLB*	288.6 (d)	Auxin efflux carrier
GA & GGA	AX-147224865	6	11297985	*AZU29N*	269.4 (d)	Geranylgeranyl reductase involved in chlorophyll a biosynthesis
CSI	AX-147228765	7	70634323	*UJVA88*	75.9 (u)	Peroxidase
				*P6RS4K*	284.5 (u)	Pentatricopeptide repeat
GA & GGA	AX-147239793	12	295929	*D1YXCG*	216.1 (d)	Ribosomal protein S5 domain 2-type fold
CSI	AX-176818776	13	115500602	*1EP8UK*	23.6 (d)	Thaumatin
AUDPC-ELS	AX-176820950	16	18466678	*8W91IE*	91.5 (d)	Superoxide dismutase
Hue	AX-176824170	19	145041727	*KVF40G*	101.2 (d)	Homeobox-leucine zipper protein
GA & GGA	AX-177643984	20	133120520	*G540IJ*	168.0 (u)	Glutaredoxin family protein

^a^Hue, hue angle; GA, green area; GGA, greener area; CSI, crop senescence index; NDVI, normalized difference vegetation index; ELS, early leaf spot; LLS, late leaf spot; AUDPC-ELS, area under disease progress curve for early leaf spot; AUDPC-LLS, area under disease progress curve for late leaf spot. ^b^Marker name. c Chromosomes. ^c^Marker position on the chromosome. ^d^Gene symbol obtained from phytozome (https://phytozome-next.jgi.doe.gov/). ^e^Position from the marker (u=upstream and d=downstream). ^f^Gene annotation obtained from phytozome.

## Conclusions

In all, ninety-seven SNPs were detected by the six multi-locus GWAS models. Out of which twenty-nine SNPs were detected by at least two models for one or more traits across 16 chromosomes with explained phenotypic variation ranging from 0.01 - 62.76%, with exception of Chr08, Chr10, Chr11, and Chr19. Two hundred and fifty-three genes were located within 300 kbp of 14 SNPs, from seventeen potential candidate genes were predicted. Most of the predicted candidate genes were found to SA, ABA, GA, auxin and MeJA responsive and MBSI CAREs implicated in plant response to biotic stresses. The results from this study would be useful for breeding leaf spots resistance cultivars.

## Data availability statement

The raw data supporting the conclusions of this article will be made available by the authors, without undue reservation.

## Author contributions

RO-F, ES, MR, DF, DO, and PO-A conducted the experiments. BK, RO-F, DP, and MBa organized and supervised the overall project. BK, YK, and ES performed the data analyses and wrote the manuscript. RO-F, DO and MBu edited the manuscript. All authors contributed to the article and approved the submitted version.

## Funding

This study was made possible by the generous support of the BMGF funded Accelerated Varietal Improvement and Seed Systems for Legumes and Cereals in Africa (AVISA) Project Grant Number OPP1198373 and the American people through the United States Agency for International Development (USAID) Cooperative Agreement No. 7200AA 18CA00003 to the University of Georgia as management entity for U.S. Feed the Future Innovation Lab for Peanut (2018–2023). The funders had no role in experiments, data analyses and publication.

## Conflict of interest

The authors declare that the research was conducted in the absence of any commercial or financial relationships that could be construed as a potential conflict of interest.

## Publisher’s note

All claims expressed in this article are solely those of the authors and do not necessarily represent those of their affiliated organizations, or those of the publisher, the editors and the reviewers. Any product that may be evaluated in this article, or claim that may be made by its manufacturer, is not guaranteed or endorsed by the publisher.

## Supplementary material

Supplementary Figure 3 Heatmap of the genomic kinship matrix obtained by the VanRaden (2008) Method among the single nucleotide polymorphism markers and 294 groundnut germplasm.
