# Supplementary material for: Multi-locus genome-wide association studies reveal genomic regions and putative candidate genes associated with leaf spot diseases in African groundnut (Arachis hypogaea L.) germplasm
Source: Front Plant Sci. 2023 Jan 5;13:1076744. doi: 10.3389/fpls.2022.1076744 (PMC9849250; doi:10.3389/fpls.2022.1076744)
Supplement: Supplementary Figure 3 — Heatmap of the genomic kinship matrix obtained by the VanRaden (2008) Method among the single nucleotide polymorphism markers and 294 groundnut germplasm. [file DataSheet_1.zip › Supplementary Figure 1.DOCX]

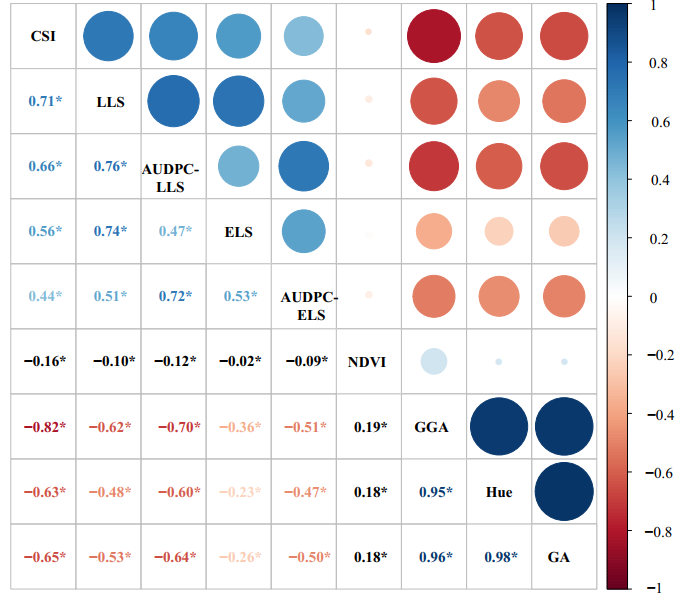


**Supplementary Figure 1:** Pearson correlation coefficients among the 8 variables for both early and late leaf spot diseases ratings. CSI= crop senescence index, LLS=late leaf spot, AUDPC-LLS = area under disease progression curve for LLS, ELS=early leaf spot, NDVI= normalized difference vegetation index, GGA=greener area, hue=hue content obtained from image, and GA= green area. Correlation coefficients with * indicate significance at *P*<0.05.
