# Supplementary material for: Multi-locus genome-wide association studies reveal genomic regions and putative candidate genes associated with leaf spot diseases in African groundnut (Arachis hypogaea L.) germplasm
Source: Front Plant Sci. 2023 Jan 5;13:1076744. doi: 10.3389/fpls.2022.1076744 (PMC9849250; doi:10.3389/fpls.2022.1076744)
Supplement: Supplementary Figure 3 — Heatmap of the genomic kinship matrix obtained by the VanRaden (2008) Method among the single nucleotide polymorphism markers and 294 groundnut germplasm. [file DataSheet_1.zip › Supplementary Figure 2.DOCX]

**
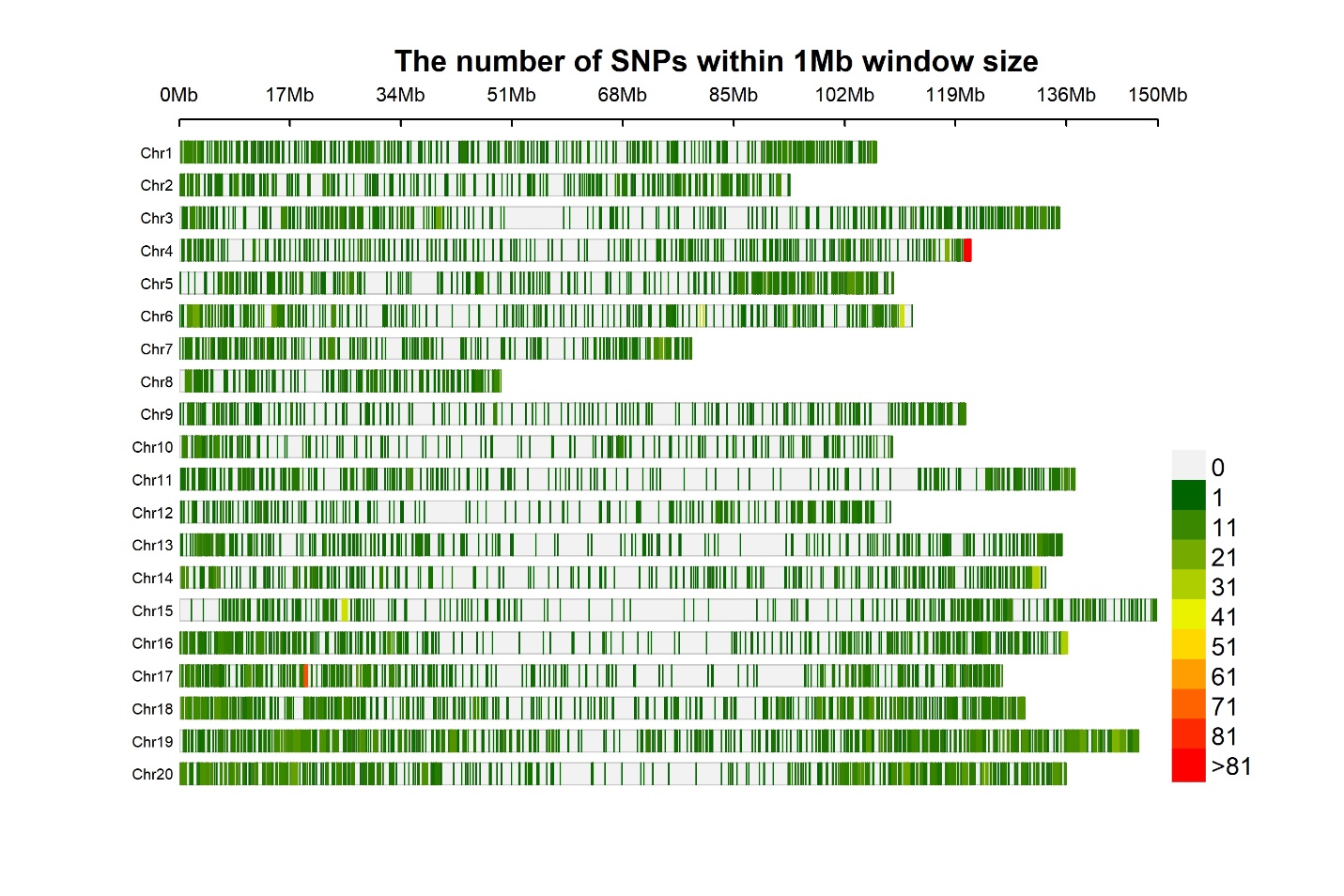
**

**Supplementary Figure 2:** Single nucleotide polymorphism marker density in the two sub-genomes (A and B). A sub-genome comprised chromosome (Chr) 1 to 10, while B sub-genome consisted of Chr11-20. The number of SNPs within 1 Mb widow size as shown the colour legend on the right hand side of the figure.
