# Supplementary material for: Multi-locus genome-wide association studies reveal genomic regions and putative candidate genes associated with leaf spot diseases in African groundnut (Arachis hypogaea L.) germplasm
Source: Front Plant Sci. 2023 Jan 5;13:1076744. doi: 10.3389/fpls.2022.1076744 (PMC9849250; doi:10.3389/fpls.2022.1076744)
Supplement: Supplementary Figure 3 — Heatmap of the genomic kinship matrix obtained by the VanRaden (2008) Method among the single nucleotide polymorphism markers and 294 groundnut germplasm. [file DataSheet_1.zip › Supplementary Table 2.DOCX]

**Supplementary Table 2:** Combined Analysis of variance (2020 and 2021) of the studied traits implemented in SAS (SAS Institute, 2010. SAS/STAT software version 9.2).

| 1. **Hue angle (Hue)** | | |  |  |  |
| --- | --- | --- | --- | --- | --- |
| **Source** | **DF** | **Sum of Squares** | **Mean Square** | **F Value** | **Pr > F** |
| **Enviro** | 1 | 231793.63 | 231794 | 3562.84 | <.0001 |
| **Rep(Enviro)** | 4 | 1208.13 | 302.03 | 4.64 | 0.001 |
| **Line** | 288 | 127479.03 | 442.64 | 6.8 | <.0001 |
| **Line*Enviro** | 275 | 31207.44 | 113.48 | 1.74 | <.0001 |
| **Error** | 1115 | 72540.42 | 65.06 |  |  |
| **Total** | 1683 | 464228.64 |  |  |  |

| 1. **Green area (GA)** | | | |  |  |
| --- | --- | --- | --- | --- | --- |
| **Source** | **DF** | **Sum of Squares** | **Mean Square** | **F Value** | **Pr > F** |
| **Enviro** | 1 | 41.47 | 41.47 | 4405.99 | <.0001 |
| **Rep(Enviro)** | 4 | 0.09 | 0.02 | 2.42 | 0.0469 |
| **Line** | 288 | 18.46 | 0.06 | 6.81 | <.0001 |
| **Line*Enviro** | 275 | 4.14 | 0.02 | 1.6 | <.0001 |
| **Error** | 1115 | 10.49 | 0.01 |  |  |
| **Total** | 1683 | 74.66 |  |  |  |

| 1. **Greener area (GGA)** | | | | | |  | |  | |  | |
| --- | --- | --- | --- | --- | --- | --- | --- | --- | --- | --- | --- |
| **Source** | | **DF** | | **Sum of Squares** | | **Mean Square** | | **F Value** | | **Pr > F** | |
| **Enviro** | | 1 | | 18.49 | | 18.49 | | 2032.61 | | <.0001 | |
| **Rep(Enviro)** | | 4 | | 0.14 | | 0.03 | | 3.83 | | 0.0042 | |
| **Line** | | 288 | | 23.76 | | 0.08 | | 9.07 | | <.0001 | |
| **Line*Enviro** | | 275 | | 4.37 | | 0.02 | | 1.75 | | <.0001 | |
| **Error** | | 1115 | | 10.15 | | 0.01 | |  | |  | |
| **Total** | | 1683 | | 56.91 | |  | |  | |  | |
| 1. **Crop senescence index (CSI)** | | | | | | |  | |  | |  |
| **Source** | **DF** | | **Sum of Squares** | | **Mean Square** | | **F Value** | | **Pr > F** | |  |
| **Enviro** | 1 | | 33914.04 | | 33914.04 | | 666.57 | | <.0001 | |  |
| **Rep(Enviro)** | 4 | | 1855.58 | | 463.89 | | 9.12 | | <.0001 | |  |
| **Line** | 288 | | 96448.43 | | 334.89 | | 6.58 | | <.0001 | |  |
| **Line*Enviro** | 275 | | 53154.81 | | 193.29 | | 3.8 | | <.0001 | |  |
| **Error** | 1115 | | 56729.19 | | 50.88 | |  | |  | |  |
| **Total** | 1683 | | 242102.05 | |  | |  | |  | |  |

| 1. **Canopy normalized difference vegetation index (NDVI)** | | | | | |
| --- | --- | --- | --- | --- | --- |
| **Source** | **DF** | **Sum of Squares** | **Mean Square** | **F Value** | **Pr > F** |
| **Enviro** | 1 | 3.96 | 3.96 | 4.55 | 0.033 |
| **Rep(Enviro)** | 4 | 3.48 | 0.87 | 1 | 0.405 |
| **Line** | 288 | 250.82 | 0.87 | 1 | 0.4811 |
| **Line*Enviro** | 275 | 246.96 | 0.90 | 1.03 | 0.3554 |
| **Error** | 1115 | 968.43 | 0.87 |  |  |
| **Total** | 1683 | 1473.66 |  |  |  |

| 1. **Early leaf spot (ELS)** | | | | | |
| --- | --- | --- | --- | --- | --- |
| **Source** | **DF** | **Sum of Squares** | **Mean Square** | **F Value** | **Pr > F** |
| **Enviro** | 1 | 1551.65 | 1551.65 | 5784.83 | <.0001 |
| **Rep(Enviro)** | 4 | 16.71 | 4.18 | 15.57 | <.0001 |
| **Line** | 288 | 339.51 | 1.18 | 4.4 | <.0001 |
| **Line*Enviro** | 275 | 321.44 | 1.17 | 4.36 | <.0001 |
| **Error** | 1115 | 299.07 | 0.27 |  |  |
| **Total** | 1683 | 2528.41 |  |  |  |

| 1. **Late leaf spot (LLS)** | | | | | |
| --- | --- | --- | --- | --- | --- |
| **Source** | **DF** | **Sum of Squares** | **Mean Square** | **F Value** | **Pr > F** |
| **Enviro** | 1 | 230.79 | 230.79 | 649.98 | <.0001 |
| **Rep(Enviro)** | 4 | 13.20 | 3.30 | 9.3 | <.0001 |
| **Line** | 288 | 663.49 | 2.30 | 6.49 | <.0001 |
| **Line*Enviro** | 275 | 272.12 | 0.99 | 2.79 | <.0001 |
| **Error** | 1115 | 395.90 | 0.36 |  |  |
| **Total** | 1683 | 1575.50 |  |  |  |

1. **Area under disease progression curve (AUDPC) – ELS (AUDPC-ELS)**

| **Source** | **DF** | **Type I SS** | **Mean Square** | **F Value** | **Pr > F** |
| --- | --- | --- | --- | --- | --- |
| **Enviro** | 1 | 641245 | 641245 | 5417.42 | <.0001 |
| **Rep(Enviro)** | 4 | 7347.76 | 1836.94 | 15.52 | <.0001 |
| **Line** | 289 | 174603 | 604.16 | 5.1 | <.0001 |
| **Line*Enviro** | 277 | 142412 | 514.12 | 4.34 | <.0001 |
| **Error** | 1124 | 133045 | 118.37 |  |  |
| **Corrected Total** | 1695 | 1098653 |  |  |  |

1. **Area under disease progression curve (AUDPC) – ELS (AUDPC-ELS)**

| **Source** | **DF** | **Type I SS** | **Mean Square** | **F Value** | **Pr > F** |
| --- | --- | --- | --- | --- | --- |
| **Enviro** | 1 | 102495 | 102495 | 645.41 | <.0001 |
| **Rep(Enviro)** | 4 | 6335.17 | 1583.79 | 9.97 | <.0001 |
| **Line** | 289 | 325692 | 1126.96 | 7.1 | <.0001 |
| **Line*Enviro** | 277 | 130421 | 470.84 | 2.96 | <.0001 |
| **Error** | 1124 | 178498 | 158.81 |  |  |
| **Corrected Total** | 1695 | 743441 |  |  |  |
