# Supplementary material for: Multi-locus genome-wide association studies reveal genomic regions and putative candidate genes associated with leaf spot diseases in African groundnut (Arachis hypogaea L.) germplasm
Source: Front Plant Sci. 2023 Jan 5;13:1076744. doi: 10.3389/fpls.2022.1076744 (PMC9849250; doi:10.3389/fpls.2022.1076744)
Supplement: Supplementary Figure 3 — Heatmap of the genomic kinship matrix obtained by the VanRaden (2008) Method among the single nucleotide polymorphism markers and 294 groundnut germplasm. [file DataSheet_1.zip › Supplementary Figure 3.pdf]

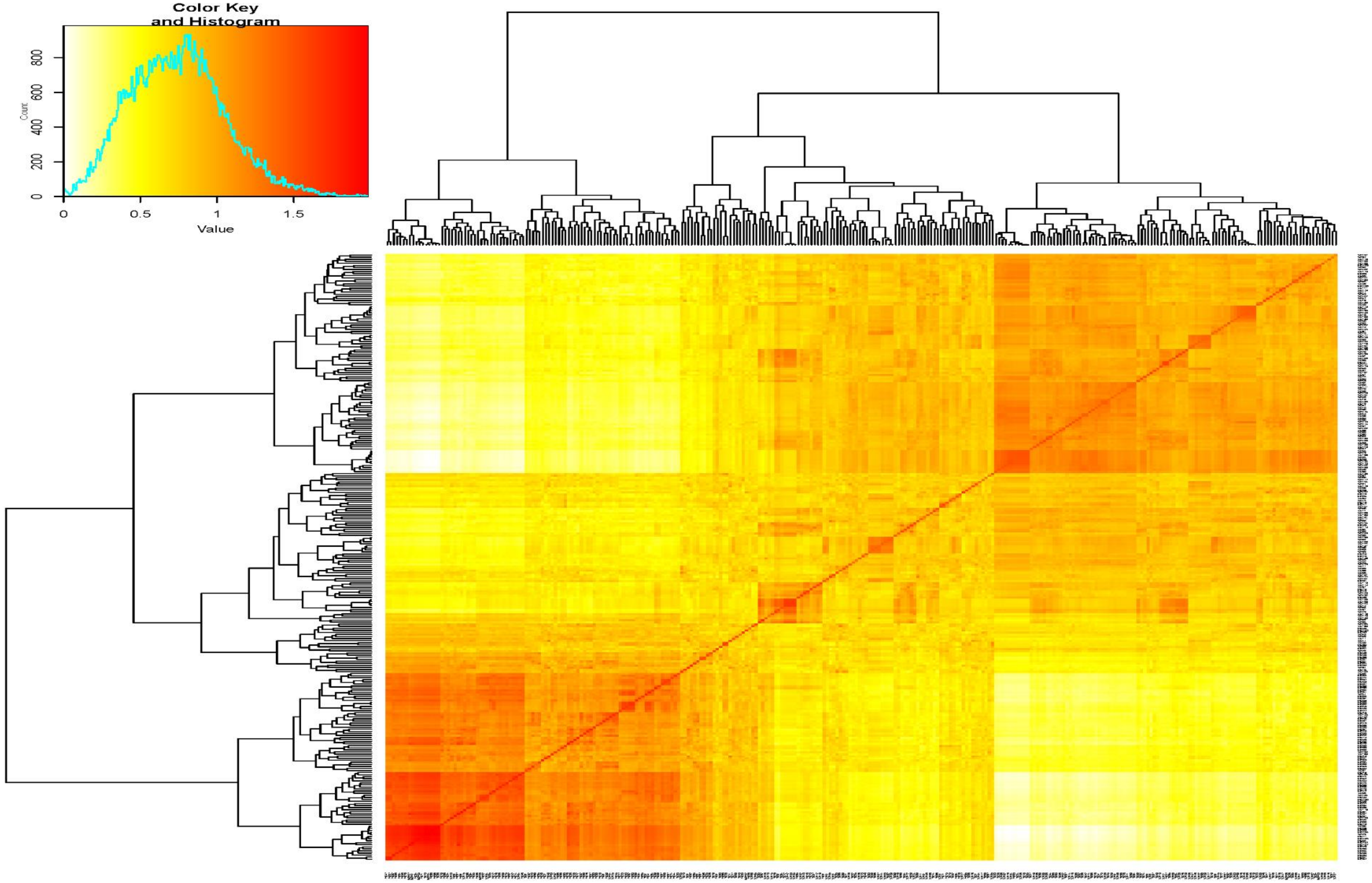

Supplementary Figure 3: Heatmap of the genomic kinship matrix obtained by the VanRaden (2008) Method among the single nucleotide polymorphism markers and 294 groundnut germplasm.

VanRaden, P. M., 2008. Efficient Methods to Compute Genomic Predictions. Journal of Dairy Science 91(11):4414-4423 doi:10.3168/jds.2007-0980.
